# Chemical Composition and Antifungal* In Vitro* and* In Silico*, Antioxidant, and Anticholinesterase Activities of Extracts and Constituents of* Ouratea fieldingiana* (DC.) Baill

**DOI:** 10.1155/2018/1748487

**Published:** 2018-11-07

**Authors:** José Eranildo Teles do Nascimento, Ana Livya Moreira Rodrigues, Daniele Silva de Lisboa, Hortência Ribeiro Liberato, Maria José Cajazeiras Falcão, Cecília Rocha da Silva, Hélio Vitoriano Nobre Júnior, Raimundo Braz Filho, Valdir Ferreira de Paula Junior, Daniela Ribeiro Alves, Selene Maia de Morais

**Affiliations:** ^1^Programa de Pós-Graduação em Ciências Veterinárias, Núcleo de Pesquisa em Sanidade Animal, Universidade Estadual do Ceará, Fortaleza, CE, Brazil; ^2^Instituto Federal do Ceará, IFCE, Campus Itapipoca, Itapipoca, CE, Brazil; ^3^Faculdade de Veterinária, Doutorado em Biotecnologia da Rede Nordeste de Biotecnologia, Universidade Estadual do Ceará, Fortaleza, CE, Brazil; ^4^Curso de Química, Universidade Estadual do Ceará, Fortaleza, CE, Brazil; ^5^Escola Adalgisa Bonfim Soares Eefm Prof, Secretaria de Educação Estado do Ceará, CE, Brazil; ^6^Faculdade de Farmácia, Universidade Federal of Ceará, Fortaleza, CE, Brazil; ^7^Universidade Christus (UNICHRISTUS), Fortaleza, CE, Brazil; ^8^Laboratório de Ciências Químicas, Universidade Estadual do Norte Fluminense, Campos dos Goytacazes, RJ, Brazil; ^9^Departamento de Química, Universidade Federal Rural do Rio de Janeiro, Seropédica, RJ, Brazil

## Abstract

*Ouratea fieldingiana* (Gardner) Engl is popularly used for wound healing. This study describes the main chemical compounds present in extracts of* O. fieldingiana* and evaluates their biological potential by investigating antifungal, antioxidant, and anticholinesterase activities. The action mechanism of main antifungal compound was investigated by molecular docking using the enzyme sterol 14-*α* demethylase, CYP51, required for ergosterol biosynthesis. The seeds and leaves were extracted with ethanol in a Soxhlet apparatus and by maceration, respectively. Both extracts were subjected to silica gel column chromatography for isolation of main constituents, followed by purification in sephadex. The structures of compounds were established by ^1^H and ^13^C-NMR spectroscopy and identified by comparison with literature data as amentoflavone and kaempferol 3-*O*-rutinoside, respectively. The antioxidant activities of the extracts were determined by the DPPH and ABTS free radical inhibition methods. In general, the extracts with the highest antioxidant activity corresponded to those with higher content of phenolic compounds and flavonoids. The ethanol extracts and two isolated compounds presented relevant antifungal activity against several* Candida* strains. The* in silico* findings revealed that the compound amentoflavone coupled with the CYP450 protein due to the low energy stabilization (-9.39 kcal/mol), indicating a possible mechanism of action by inhibition of the ergosterol biosynthesis of* Candida* fungi.

## 1. Introduction

This work investigates the therapeutic potential of* Ouratea fieldingiana*, a shrub of the Ochnaceae family, found mainly in Ceará and Rio Grande do Norte states (Brazil). This family presents tropical and subtropical characteristics, with arboreal, shrubby, and rare herbaceous representatives, including about 40 genera and 600 species.

The oil of* O. fieldingiana* seeds, extracted by decoction, is popularly used for healing skin wounds [1]. The oil obtained by extraction with hexane from* O. fieldingiana* seeds presented antibacterial and antifungal activities [2]. The results of another study showed that the anti-inflammatory activity* O. fieldingiana* seed oil was also associated with the presence of phenolic compounds and fatty acids [3]. The treatment with* Ouratea *sp. seed oil showed a collagen effect, which may be associated with the high levels of omega-6 and omega-9. Finally, [4] concluded that* Ouratea* sp. oil has good therapeutic potential in a model of cutaneous wound healing.

Plants of the* Ouratea* genus contain several types of flavonoids, like flavones, flavonols, isoflavones, chalcones, and anthocianins in the form of monomers, glycosides, bi- or bisflavonoids, like hexaspermone, amentoflavone, agatisflavone, robustaflavone, and lanaraflavone. The biflavonoid 7′′-O-methylagatisflavone (from* O. hexasperma*), amentoflavone (from* O. semiserrata*), and the acetylated derivative of amentoflavone presented DNA inhibitory activity to poisomerase type I, and potent inhibition of the growth of Ehrlich carcinoma cells [5].

Alzheimer's disease is neurodegenerative and has a strong socioeconomic impact, being associated with neurotransmitter deficits in the brain [6]. A known treatment for this disease is restoration of the cholinergic function, for which compounds that inhibit the acetylcholinesterase enzyme (AChE) improve the content of the neurotransmitter acetylcholine [7]. Then the oil and extracts of* O. fieldingiana* were also tested for inhibitory activity of acetylcholinesterase (AChE), aiming for new compounds to fight against Alzheimer's disease.

The wound healing action is closely associated with the antimicrobial [8] and antioxidant [9] activities of medicinal plants, so in this study several extracts and constituents of* O. fieldingiana* were evaluated as antioxidant and antifungal activities against* Candida* strains.

It is estimated that* Candida *infections (*Candida albicans*,* Candida parapsilosis*,* Candida glabrata*,* Candida tropicalis*, and* Candida krusei*) [10] correspond to 80% of the fungal infections detected in the urinary and blood flow of patients, as well as in the surgical environment [11]. Polyenes, flucytosine, and azole drugs are the most recommended for the treatment of* Candida* infections [12]. Some drugs with antifungal properties have inhibited the enzyme sterol 14-*α* demethylase, CYP51 [13], which is required for the biosynthesis of ergosterol, essential for the fungal membrane maintenance [14]. Thus, recently* in silico* studies examining the drug-protein interaction by the molecular docking method [15] have become a powerful tool to characterize possible compound activity in a given protein [16].

## 2. Materials and Methods

### 2.1. General Experimental Procedures

The isolation of compound was performed in an open column chromatography (60 cm length and 3 cm diameter) using silica gel 60 (60–120 mesh size, Merck) as stationary phase, being eluted with solvents hexane, chloroform, ethyl acetate, and methanol in mixtures of increasing polarity. The compounds were visualized by UV detection and/or sprayed with a solution of vanillin/sulphuric acid/EtOH. The 1D and 2D NMR data were acquired with a Bruker Avance DPX-500 spectrometer. Chemical shifts, given on the *δ* scale, were referenced to the residual undeuterated portion of the deuterated solvent CDCl_3_.

### 2.2. Plant Material

Leaves, branches, and seeds of* O. fieldingiana *collected in the city of Itapipoca, Ceará, Brazil, were used. An exsiccate with plant parts was prepared and deposited in the Prisco Bezerra Herbarium of the Federal University of Ceará, under number 57817.

### 2.3. Preparation of Ethanol Extracts with Leaves and Branches of* O. fieldingiana*

The plant material was collected and dried in the sun. After drying, 1 kg of leaves and 590 g of branches were obtained, which were shredded in a domestic multiprocessor. The leaves were macerated with 10 L of ethanol PA 96° GL, for seven days at room temperature; then the solution was evaporated under reduced pressure (40 rpm at 60°C) leaving the leaf ethanol extract, LEE (50.82 g). Also, 590 g of branch was placed in 5 L of 96% ethyl alcohol to obtain the branch ethanol extract, BEE (43.43 g), by the same procedure. The LEE and BEE were passed through a filtration column and eluted with the solvents hexane, chloroform, ethyl acetate, and methanol to obtain the respective fractions, LEE (HF: hexane fraction), LEE (CF: chloroform fraction), LEE (EAF: ethyl acetate fraction), LEE (MF: methanol fraction); BEE (HF: hexane fraction), BEE (CF: chloroform fraction), BEE (EAF: ethyl acetate fraction), and BEE (MF: methanol fraction).

### 2.4. Preparation of O. fieldingiana Seed Extracts

The crushed seeds were submitted to extraction with hexane and then ethanol in a Soxhlet apparatus, obtaining the respective hexane (SHE) an ethanol extracts (SEE).

### 2.5. Determination of the Total Phenol Content of the Leaf, Branch, and Seed Extracts of* O. fieldingiana*

Extracts samples (7.5 mg) were dissolved in 25 mL of methanol and 100 *μ*L aliquots were taken for analysis by the Folin-Ciocalteu method, which is based on the oxyreduction reactions between the phenolic compounds and metal ions, causing the formation of a blue complex, which is absorbed at 750 nm [17]. A standard curve was prepared with gallic acid. Indicate formula used in the calculation of total phenol content and total flavonoid content. The equation for the calibration curve of gallic acid was Y = 0.0013X - 0.018, where X is the concentration of gallic acid, Y is the absorbance at 750 nm, and the correlation coefficient R = 0.998. All analyses for calculations of total phenol content were performed in triplicate. The results were analyzed by Microsoft Excel 2010 [18].

### 2.6. Determination of the Flavonoid Content of Leaf, Branch, and Seed Extracts* O. fieldingiana*

Quantification of the flavonoid content of extracts (at the concentration of 2 mg/mL) was performed using 2% aluminum chloride in methanol in a spectrophotometer, with readings at 425 nm. A standard curve was prepared with quercetin. The quercetin calibration curve equation was Y = 0.04215X - 0.0118, where X is the quercetin concentration, Y is the absorbance at 425 nm, and the correlation coefficient R = 0.996. All analyses for calculations of flavonoid content were performed in triplicate. This test was performed in triplicate and followed the method proposed by [19].

### 2.7. Determination of Antioxidant Activity of Leaf, Stem, and Seed Extracts of* O. fieldingiana*

#### 2.7.1. By the DPPH Method

Several solutions of the extracts of* O. fieldingiana* were prepared in the following concentrations: 250, 125, 25, 12.5, 1.25, 0.25, 0.125, and 0.025 *μ*g/mL. The negative control was a DPPH methanol solution and the positive control was prepared by mixing a standard (quercetin) and DPPH. Methanol solutions of the extracts (100 *μ*L) were mixed with 3.9 mL of a DPPH solution; then the solutions were stored in the dark for 60 minutes and the reading was performed by spectrophotometer at the wavelength of 515 nm [18, 20]. The DPPH free radical inhibition was calculated by the scavenging index (SI_50_) = (Abs_DPPH_ – Abs_Sample_) x 100 / Abs_Sample_.

#### 2.7.2. By ABTS Method (2,2′-Azinobis-(3-ethylbenzothiazoline-6-sulfonic Acid)

The ABTS^+•^ solution (7 mM, 5 ml) was mixed with 88 *µ*l of potassium persulfate (140 mM). The mixture was shaken and kept in the dark at room temperature for 16 h. Then, 1 ml of this solution was added to 99 ml of ethanol. The absorbance was read at 734 nm (0.715). Several solutions of decreasing concentrations of plant extracts (10000 to 5 *μ*g/mL) were prepared and 3.0 ml of an ABTS^+•^ solution was added to 30 *μ*l of these solutions, and after 6 min readings were taken at 734 nm [21]. In order to evaluate the radical scavenging activity, the percentage of inhibition was obtained according to the following equation: IP% inhibition = (Abs_ABTS_ – Abs_Sample_) x 100 / Abs_ABTS_

The effective concentration of the antioxidant required to decrease the initial ABTS concentration by 50% (EC_50_) was calculated through a linear regression curve plotted with Excel. To plot the points, the values of the means obtained from triplicates were used for each of the tests.

### 2.8. Isolation and Characterization of the Constituents of the Leaf and Seed Ethanol Extracts of* O. fieldingiana*

The extracts from the leaves and seeds were passed through a vacuum-filter chromatographic column and eluted with solvents of increasing polarities: hexane, chloroform, ethyl acetate, and methanol. Fractions were obtained and compared by thin layer chromatography (CCD) for the isolation of the constituents. The isolated compounds were subjected to spectroscopic analyses, mainly ^1^H and ^13^C-NMR nuclear magnetic resonance and mass spectrometry, to determine the chemical structures

### 2.9. Qualitative Determination of Acetylcholinesterase Inhibitory Activity

The bioassay consisted of application of the samples in TLC plates, which were prepared by mixing of 5,5-dithiobis-2-nitrobenzoic acid (DTNB or Ellman's reagent) and a buffer solution of acetylthiocholine iodide (ATCI). Subsequently, the AChE enzyme was sprayed and after 3 min the presence of spots (halos) was observed and measured (in mm) on the yellow plate [22, 23]. Physostigmine was used as a positive control because it is the best chemical substance to inhibit acetylcholinesterase.

### 2.10. Quantitative Determination of Acetylcholinesterase Inhibitory Activity

The anticholinesterase activity was quantitatively measured using a Biotek ELISA microplate reader (model ELX 800 with Gen5 V2.04.11 software), based on the method described by [16], as modified by [24].

### 2.11. Antifungal Susceptibility Tests

The extracts SHE, SEE, LEE, LEE (FM), BEE, and BEE (AF) were tested against two fungal strains from the culture collection,* Candida parapsilosis* (ATCC® 22019™) and* Candida krusei* (ATCC® 6258™), and two other fluconazole-resistant strains, from the Laboratory of Bioprospection and Experimentation of Yeasts of Federal University of Ceará (LABEL). These were seeded in Sabouraud dextrose agar and incubated at 35°C for 24 h. In the antifungal tests with amentoflavone and kaempferol 3-*O*-rutinoside, three strains from the collection were used*: Candida albicans *(ATCC® 14053™)*, Candida parapsilosis* (ATCC® 22019™), and* Candida krusei* (ATCC® 6258™), as well as one clinical fluconazole-resistant strain. These were also seeded in Sabouraud dextrose agar and incubated at 35°C for 24 h.

The microdilution method was used in accordance to the document M27-A3 [25], using the culture medium RPMI 1640 (pH 7.0 ± 0.1) buffered with 0.165 M of morpholinepropanesulfonic acid (MOPS) (Sigma, USA). All samples were prepared in dimethyl sulfoxide (DMSO) (Sigma, USA) in a maximum 2% proportion to avoid interference in the microorganism structure. The extracts SHE, SEE, LEE, LEE (FM), BEE, and BEE (AF) were tested in concentrations ranging from 1000 to 1 *µ*g/mL and the compounds amentoflavone and kaempferol 3-*O*-rutenoside in the range from 500 to 0.97 *µ*g/mL.

Compounds were tested together to ascertain synergism at a concentration range from 2.5 to 0.0049 mg/mL (1:1). An initial inoculum suspension was prepared from 24 h culture of the yeasts to be tested, adjusted to 0.5 on the McFarland scale using Sabouraud dextrose agar. Serial dilutions were then prepared in RPMI 1640 medium to obtain final inocula containing 0.5 to 2.5 x 10^3^ CFU / mL. The microplates were incubated for 24 h at 35°C (± 2°C). The readings were performed visually as recommended by the Clinical and Laboratory Standards Institute (CLSI).

The minimum inhibitory concentration (MIC) was determined as the lowest concentration of the drug capable of inhibiting 50% and 100% growth of the microorganism, for the tests of the substances SHE, SEE, LEE, LEE (MF), BEE, and BEE (MF) compared to the control also containing only the culture medium and the standardized inoculum [25]. The tests were performed in triplicate.

For the tests of the isolated compounds, the minimum inhibitory concentration (MIC) was determined as the lowest concentration of the drug capable of inhibiting 50% growth of the microorganism, compared to that in the control also containing only the culture medium and the standardized inocula [25]. The tests were performed in triplicate.

### 2.12. In Silico Analysis of the Properties of Amentoflavone

The geometric arrangement of a given molecule can present structural similarity with other compounds already identified, establishing a correlation in biological activity [26]. Therefore, the structural data of the amentoflavone molecule were obtained from the PubChem database [27] for analysis of the structural similarity by comparison with the DrugBank database, with similarity limit, ST: 0.7 [28]. Then a three-dimensional optimization of the conformation of the amentoflavone was prepared using the MarvinSk program [29], with valence checking and geometric analysis of the molecule, to minimize the steric hindrance, and the energy minimization was performed by the MMFF94 force field [30]. Finally, the theoretical LD_50_ of the amentoflavone was calculated by the ProTox server [31], and the toxicity of the compound was predicted using the Toxin-Toxin target database (T3DB), which presents 3,673 toxins [32].

### 2.13. Obtaining the* Candida *CYP51 Protein Molecule and Optimization of Its Spatial Structure

The protein* Candida albicans*, CYP51 (id: 5v5z), used as a molecular target in the computational simulations, was obtained from the Protein Database, PDB [33], and was edited to remove the water molecules around the surface of the protein and to add polar hydrogen atoms by the PyMOL 2.0 program [34].

#### 2.13.1. Molecular Redocking and Docking of Itraconazole Coupled to C. albicans Protein

The itraconazole linked to the catalytic site of the* C. albicans* CYP51 (PDB: 5v5z) protein was removed by the PyMOL 2.0 program and then redocked in the autodock Vina, serving as reference for the docking. The molecular docking of amentoflavone was carried by addition Kollman charges [35], using AutoDockTools 1.5.6 [36]. Also, the chosen program used the protein as input, whereas the amentoflavone molecule was in the flexible form, having greater freedom regarding torsion angles in the search for a favorable conformation [37]. In this way, a grid box was created respecting the protein group HEME (C_34_ H_32_ Fe N_4_ O_4_), around the active site of the protein with coordinates x: -43.454; y: -13.913; z: 23.332, in AutoDockTools 1.5.6 [36]. After the execution of the program, the molecules with the best position within the active site of the protein were chosen, with lower binding energy found in kcal/mol (score = -ΔG) [38], and the root mean square deviation (RMSD) was calculated [39], using the VMD program [40].

### 2.14. Statistical Analysis

For each parameter evaluated, the mean ± standard deviation (SD) was calculated. In the case of multiple comparisons between groups, the homogeneity of the variables involved was tested through the Bartlett test. When homogeneity was observed between the variables, analysis of variance (ANOVA) was applied, followed by the Tukey test. All conclusions were taken at the significance level of 5% (p < 0.05). The tests were performed in triplicate and the values expressed as mean ± standard deviation. The experiment was completely randomized, where the extracts were the treatments.

In the antifungal test, experiments measuring the susceptibility of compounds and the expression of synergism profiles* in vitro* were performed in triplicate. The geometric mean of the three trials was used to compare the MIC_50_ results statistically.

## 3. Results

### 3.1. Structural Characterization of Chemical Constituents

The chromatographic treatment of the seed ethanol extract led to the isolation of a compound identified by spectral data in comparison to literature data [41, 42] as kaempferol 3-*O*-rutinoside, not previously reported in this plant. The ^1^H-NMR spectra of this compound showed several peaks in the aromatic region from 6.21 to 8.06 ppm and hydrogens of two sugar units with a signal at 5.12 ppm and another at 4.51 ppm, characteristic of glucose and rhamnose anomeric hydrogen, respectively.

**Figure 1:**
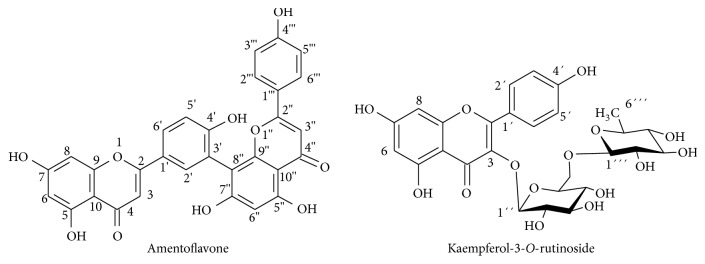
Chemical representation of compounds present in* Ouratea fieldingiana*.

The methanol fraction of the leaf ethanol extract was chromatographed in a silica gel column and a compound was isolated, whose chemical structure was established by ^1^H and ^13^C-NMR analyses. The ^1^H-NMR of this compound showed several peaks in the aromatic region from 6.29 to 8.41 ppm and the ^13^C-NMR spectrum displayed 30 signals in the Csp^2^ region, revealing the dimeric flavonoid characteristic. By comparison with literature data [41, 42] it was characterized as amentoflavone, a biflavonoid present in other* Ouratea* species [5]. The chemical structures of two isolated compounds are displayed in Figure 1.

The complete spectral assignments of hydrogens and carbons are shown below.

*Amentoflavone Assignments.* ^1^H NMR (MeOD, 300 MHz): *δ* 6.60 (s, H-3), 6.16 (d, 2.0, H-6), 6.22 (d, 2.0, H-8), 8.14 (d, 2.2, H-2'), 7.09 (d, 8.6, H-5'), 7.87 (dd, 8.6, 2.2, H-6'), 6.29 (s, H-6”), 7.59 (d, 8.7, H-2”'), 6.64 (d, 8.7, H-3”'), 6.64 (d, 8.7, H-5”'), 7.59 (d, 8.7, H-6”').

^13^C NMR (MeOD, 75 MHz): *δ* 165.11 (C-2), 102.23 (C-3), 182.36 (C-4), 161.67 (C-5), 98.90 (C-6), 164.95 (C-7), 93.80 (C-8), 155.18 (C-9), 103.78 (C-10), 120.61 (C-1'), 131.40 (C-2'), 122.31 (C-3'), 161.06 (C-4'), 118.25 (C-5'), 126.68 (C-6'), 164.21 (C-2”), 101.80 (C-3”), 182.58 (C-4”), 157.95 (C-5”), 100.96 (C-6”), 167.71 (C-7”), 106.16 (C-8”), 161.54, (C-9”), 103.04 (C-10”), 121.89 (C-1”'), 127.90 (C-2”'), 115.38 (C-3”'), 160.91 (C-4”'), 115.38 (C-5”'), 127.90 (C-6”') [42].

*Kaempferol-3-O-Rutinoside NMR Assignments.* ^1^H NMR (MeOD_,_ 300 MHz): *δ* 6.21 (1H, d, J = 1.9, H-6), 6.41 (1H, d, J = 1.9, H-8), 8.06 (2H, d, J = 8.8, H-2′,6′), 6.88 (2H, d, J = 8.8, H-3′,5′), 5.12 (1H, d, J = 7.2, Glc H-1), 4.51 (1H, s, Rha H-1), 0.88 (3H, s, Rha-CH_3_), 3.25–3.82 (other H). ^13^C NMR (CDCl_3,_ 75 MHz) [41].

^13^C NMR (MeOD, 75 MHz): *δ* 159.58 (C-2), 135.68 (C-3), 179.57 (C-4), 163.15 (C-5), 100.15 (C-6), 166.16 (C-7), 95.09 (C-8), 158.71 (C-9), 105.85 (C-10), 121.00 (C-1'), 132.51 (C-2'), 116.30 (C-3'), 161.62 (C-4'), 116.30 (C-5'), 132.51 (C-6'), 104.77 (C-1”), 74.07 (C-2”), 78.32 (H-3”), 71.62 (H-4”), 78.05 (H-5”), 68.74 (H-6”), 102.58 (H-1”'), 72.25 (H-2”'), 72.49 (H-3”'), 73.62 (H-4”'), 69.89 (H-5”'), 18.05 (H-6”', CH_3_) [42].

### 3.2. Determination of the Total Phenol and Flavonoids Content and Antioxidant Activity of Extracts of Leaves, Branches, and Seeds of* O. fieldingiana, *Table 1.

Regarding phenolic content, LEE, LEE (MF), BEE, and BEE (EAF) showed the best results. In relation to flavonoid content, the extracts LEE and LEE (MF) were better. In general, the more polar extracts had higher yields of phenolic compounds.

To evaluate the antioxidant activity of the samples, two different methods, using DPPH^.^ and ABTS^+.^ radicals, were performed. In the DPPH^.^ test, the IC_50_ (concentration capable of inhibiting the radical by 50%) was evaluated and better results were shown by LEE, LEE (MF), BEE, and BEE (FEA).

In relation to the ABTS^+.^ method, the extracts BEE, BEE (MF), BEE (EAF), LEE, and LEE (MF) presented IC_50_ values similar to the standard quercetin. Like the more polar extracts, which presented higher yield of phenolic compounds, these also presented higher antioxidant activities according to both methods.

### 3.3. Acetylcholinesterase Enzyme Inhibition Test by ELISA

All fractions were submitted to the quantitative anti-acetylcholinesterase test to find the IC_50_ and the results of* O. fieldingiana* fractions are shown in Table 2. The leaf ethanol extract showed the best anticholinesterase action, similar to physostigmine, the alkaloid standard. The biflavonoid amentoflavone is present in the leaf ethanol extract, but the extract showed superior results probably due to the synergism among the main constituents, Table 2.

### 3.4. Antifungal Tests

In general, the extracts demonstrated antifungal activity against all isolates except for EHS, and especially against ATCC strains, because resistant strains were less active, Table 3.

Among the isolated compounds, amentoflavone was more active against fungal isolates than kaempferol-3-O-rutinoside, but the mixture of the two constituents revealed synergism since MIC 50 values were lower against all strains tested, Table 4.

#### 3.4.1. *In Silico* Analysis of the Properties of Amentoflavone

**Figure 2:**
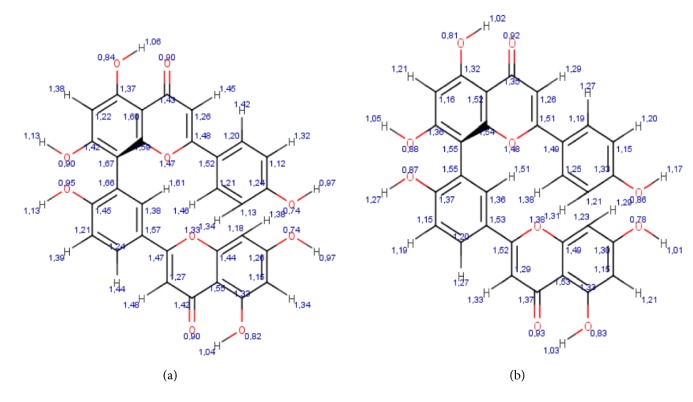
Optimization of the geometry of the amentoflavone molecule. (a) Before energy minimization by the MMFF94 force field. (b) After energy minimization by the application of the force field MMFF94.

The atomic coordinates of the amentoflavone molecule, obtained from PubChem (CID 5281600), had no valence error. In the geometric analysis, the molecule was optimized by MMFF94 energy = 163.74 kcal/ mol, minimal projection area = 74.63 Å^2^, maximal projection area = 126.50 Å^2^, minimal projection radius = 6.79 Å, maximal projection radius = 9.39 Å, length perpendicular to max area = 10.03 Å, length perpendicular to min area = 18.40 Å, van der Waals volume = 432.58 Å^3^, for improved geometry: MMFF94 energy = 76,24 kcal/ mol, minimal projection area = 62.78 Å^2^, maximal projection area = 133.27 Å^2^, minimal projection radius = 6.16 Å, maximal projection radius = 8.65 Å, length perpendicular to max area = 9.33 Å, length perpendicular to min area = 15.58 Å, and van der Waals volume = 441.42 Å^3^, Figure 2.

In the analysis of the structural similarity by the DrugBank server, amentoflavone (C_30_H_18_O_10_) resembled the following molecules: (DrugBank-DB02375) - myricetin (C_15_H_10_O_8_); (DrugBank-DB04216) - quercetin (C_15_H_10_O_7_); (DrugBank-DB07352) – apigenin (C_15_H_10_O_5_); (DrugBank-DB07795) - 3,7,3′,4′ tetrahydroxyflavone (C_15_H_10_O_6_); (DrugBank-DB08230) - 5,7-dihydroxy-2-(3,4,5-trihydroxyphenyl)-4H-chromen-4-one (C_15_H_10_O_7_); (DrugBank-DB11259)- diosmetin (C_16_H_12_O_6_); (DrugBank-DB12672) -icaritin (C_21_H_20_O_6_). In the toxicity study, amentoflavone obtained an LD_50_ of 3919mg/kg, being classified in category 5 according to the globally harmonized system of classification of labeling of chemicals (GHS).

#### 3.4.2. Molecular Redocking and Docking

**Figure 3:**
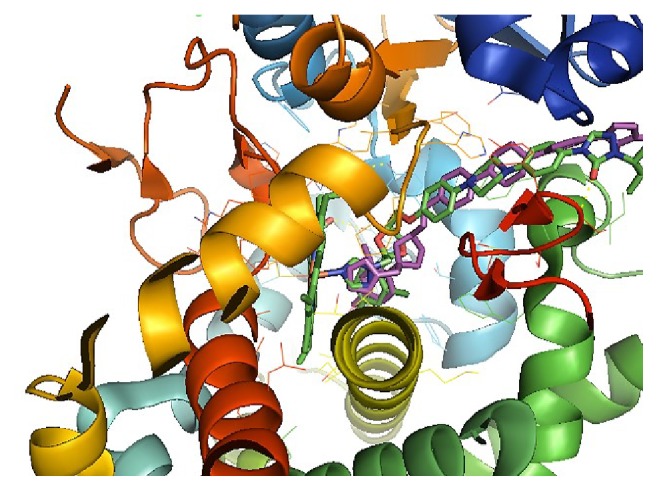
Overlap of the itraconazole binders to the catalytic site of the protein CYP51.

A valuable alternative for the study of drug-protein interaction is the molecular docking technique, which evaluates the behavior of a given molecule within the active site of the protein [43]. Therefore, in the simulation of the molecule redocking, the itraconazole pose was obtained at the active site of the CYP51 protein with the lowest binding free energy of -12.73 kcal/mol, with the approximation of the itraconazole pose in the native conformation of the protein CYP51, being observed in the overlap of the links, lower root mean square deviations, RMSD > 2.0 Å [44], Figure 3.

**Figure 4:**
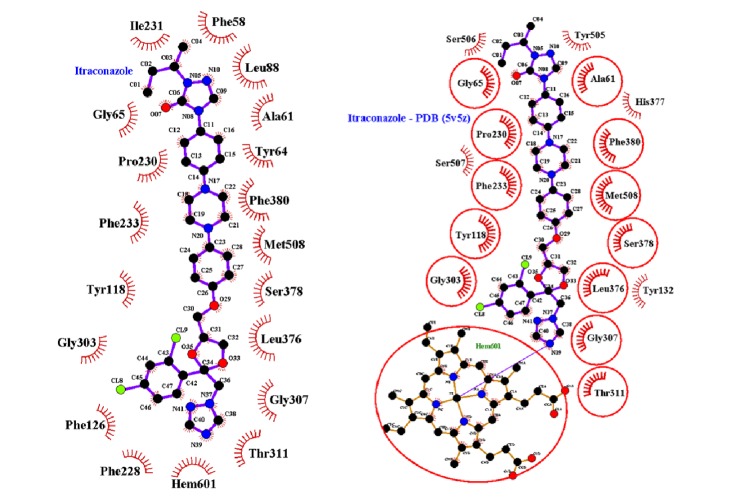
Conformation of compound submitted to the computational test. (Itraconazole) Pose obtained from re-docking molecule. (PDB-5v5z) Itraconazole complexed the CYP51 protein in its native conformation.

The residues involved in the interaction were Gly65(A); Pro230(A); Phe233(A); Ala61(A); Phe380(A); Met508(A); Ser378(A); Leu376(A); Tyr188(A); Gly303(A); Gly307(A); and Thr311(A), along with the Hem601(A) group, as shown in Figure 4.

**Figure 5:**
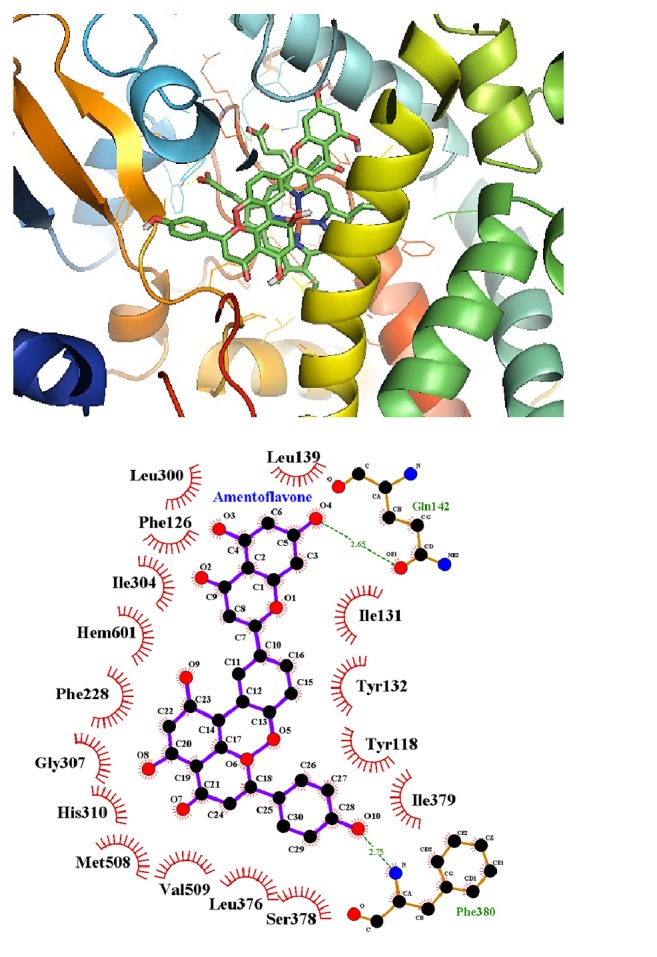
Molecular docking of amentoflavone with the CYP51 protein, characterizing the residues involved in the hydrophobic interaction and hydrogen bond.

The amentoflavone molecule coupling with the CYP51 protein, and the spatial conformation with the lowest free energy of -9.39 kcal/mol, showed 15 binding residues (Ser378(A); Leu376(A); Val509(A); Met508(A); His310(A); Gly307(A); Phe228(A); Ile304(A); Phe126(A); Leu300(A); Leu139(A); Ile131(A); Tyr132(A); Tyr118(A); Ile379(A)), with the group HEME (Hem601(A)), performed hydrophobic interactions, and also two hydrogen bonds with residues: Gln142(A), with a distance of 2,65 Å, and Phe380(A), with a distance of 2.75 Å, with a root mean square deviation (RMSD) >2.0 Å [44], as shown in Figure 5.

## 4. Discussion

The wound healing action found for the* Ouratea* sp. seed oil displays a collagen effect, attributed to the presence of w-6 and w-9 fatty acids [4]. The main fatty acid in* O. fieldingiana* was oleic (43.06%), a w-9 fatty acid, which can contribute to the healing action. In another work, the effect of the anti-inflammatory activity* O. fieldingiana* seed oil was also associated with the presence of phenolic compounds besides fatty acids [3]. The activity of antioxidant compounds in a mixture depends on several physicochemical factors, like the interactions among other antioxidant compounds and with other constituents, such as fatty acids linked to phospholipids or triacylglycerides. Therefore, an individual antioxidant constituent could give limited results when associated with other constituents in a plant extract [45]. The seed oil of* O. fieldingiana* is also popularly used for wound healing on the skin. This action can be related to the presence of phenolic compounds with antioxidant and antifungal properties, besides the presence of unsaturated fatty acids.

Plant derived products have been used to treat many diseases worldwide and are still playing a major role in healthcare. It is known that the wound-healing process can be aided by the presence of antioxidants. The presence of effective antioxidants in various plant extracts is well known and many plant extracts or plant-derived compounds possessing high antioxidant properties also show wound-healing activities [3, 9].

The glycosylated flavonoid nicotiflorin (kaempferol-3-*O*-rutinoside), extracted from the seeds, and amentoflavone, a biflavonoid obtained from the ethanol extract of the leaves of* O. fieldingiana*, displayed antioxidant activity, as previously reported [46]. In that study, the authors showed the antioxidant activity of several flavonoids and their rutinoside derivatives, O-glycosides, and dimers, deducing that the higher the number of hydroxyls, the greater the antioxidant activity. However, the 3′,4′-dihydroxy system, present in quercetin, used as standard in the study of* O. fieldingiana*, probably confers greater antioxidant activity on the molecule.

Infected wounds heal less rapidly, so there is a need to stimulate the healing process and restore the normal functions of the affected part of the body, preventing infection and activating tissue repair processes. Antibacterial and healing compounds in a traditional remedy can induce this occurrence and may be beneficial in treating wounds [8].

The oil from the seeds of* O. fieldingiana* fruits presented antibacterial and antifungal activities [2]. Our study corroborated this action of the oil and extends it to the leaf and branch extracts and the constituents amentoflavone and kaempferol 3-*O*-glucoside, isolated from the leaves and the seeds, respectively, which also display antimicrobial activities, a useful characteristic in the wound healing process. All the extracts and compounds tested showed antifungal activity within the tested range (1000 to 1.95 *μ*g/mL) against all isolates, except for the hexane seed extract (SHE). However, the active extracts showed selectivity for the* C. krusei* isolate. The substances amentoflavone and kaempferol 3-O-rutinoside used in combination showed superior antifungal activity than the extracts alone.

On the other hand, in the study of drug-protein interaction with the molecular coupling method, it was observed in the simulation of the molecular redocking of the ligand (Itraconazole) with the CYP51 protein of* Candida albicans* that coupling was established at the catalytic site of the protein (-12.73 kcal/mol), with a high level of similarity to the CYP51 native structure site, demonstrating reliability in the coupling simulations [47] (Figure 2). Furthermore, in the coupling simulation of amentoflavone with the CYP51 protein, it was observed that this compound showed favorable interactions with several residues of the active site of the protein and the group (Hem601). This result demonstrates that amentoflavone is a strong candidate as an antifungal drug, when compared to the Irfan and Abid results [48], where the molecular fit with 18 synthetic antifungal triazole derivatives in the CYP51 protein of* Candida albicans* showed a free energy range of -9.8 to -7.4 kcal/mol, including the antifungal fluconazole (-8.6 kcal/mol). Moreover, in the present study, low binding energy (-9.39 kcal/mol) was obtained for amentoflavone, CYP51, near to that of fluconazole, which is considered by ANVISA as a drug of choice for the treatment of fungal infections (especially vaginal candidiasis caused by* Candida* species).

Reactive oxygen species (ROS) are directly involved in the pathogenesis of many diseases and the antioxidants from* O. fieldingiana *act by attenuating the cellular oxidative damage. In this respect, free-radical scavenging was observed in experimentally induced liver injuries [49].

The search for plants that have antimicrobial and antioxidant properties can lead to relatively nontoxic and cost-effective antifungal products [50]. So, using substances with antioxidant properties is a useful way to achieve this goal, since in general good antioxidant plant extracts contain phenolic compounds like flavonoids and organic acids that also display antimicrobial activity. Seeds, leaves, and branches of* O. fieldingiana* were submitted to maceration with several solvents to prepare the respective extracts. These extracts were treated in silica gel chromatographic columns and two flavonoids were isolated. The structure was elucidated by NMR analysis and amentoflavone and kaempferol 3-*O*-rutinoside were identified.

Biflavones, including amentoflavone, have been reported mainly in leaves of various* Ouratea* species [51]. Kaempferol 3-*O*-rutinoside is an antioxidant found in fruits and vegetables that fights free radicals, which promote the development of cancer, besides being a potent promoter of apoptosis [52]. This substance is much less toxic to normal cells than standard chemotherapy drugs [53].

The extracts of* O. fieldingiana* showed excellent antioxidant activity. The standard used was quercetin, a compound that belongs to the class of the most common flavonoids and stands out for its great antioxidant potential. The extracts with the best antioxidant activity were BEE (EAF) and LEE (MF), those containing the largest amount of phenolic compounds. The good antioxidant activity presented by the extracts can be correlated to the presence of flavonoids and other phenols, present in all samples analyzed.

The results found in this study indicate that this species has antioxidant chemicals capable of capturing free radicals. These substances are promising for studies aimed at preventing diseases due to oxidative stress. However, the antioxidant tests performed do not allow a precise definition of the antioxidant effects because they are* in vitro. In vivo* study is required to determine whether this medicinal plant can be used effectively for this purpose [45].

Oxidative damage is considered to be one of the most important mechanisms involved in the pathogenesis of Alzheimer's disease, which results in the chemical modification of the biological molecules, leading to neuronal death. It has been reported that plants containing vitamins (C, E, carotenoids, etc.), flavonoids (flavones, isoflavones, flavonones, anthocyanins, and catechins), and polyphenols (ellagic acid, gallic acid, and tannins) possess remarkable antioxidant activities [54].

Other studies have shown that the antioxidant capacity of vegetable oils can be influenced by the concentration of some tocopherols, such as *γ*- and *δ*-tocopherols, or phenolic compounds [3, 55]. In a study carried out by [4], the results of the chemical evaluation (by gas chromatography together with mass spectrometry) of the fixed oil of* Ouratea* sp. detected the major components as being the unsaturated fatty acids linoleic acid (40.88%) and oleic acid (28.29%), along with palmitic acid as the saturated fatty acid (20.65%).

The anticholinesterase action of plant extracts in combination with antioxidant properties can also contribute to complementary therapy for Alzheimer's disease.

The inhibition of acetylcholinesterase by the ethanol extract of the* O. fieldingiana *leaves was excellent, with IC_50_ of 0.81*μ*g/mL ± 0.004, being statistically equal to the physostigmine standard, followed by the ethanol extracts of the seeds and branches and isolated compounds. Compared to eserine, another standard used as AChE inhibitor, almost all samples presented activity. Amentoflavone, isolated from the root extract of* Cnestis rustina*, demonstrated antidepressant and anxiolytic effects [56, 57], so our findings confirm the potential of this substance for nervous system problems.

## 5. Conclusion

*O. fieldingiana* is a good source of flavonoids, biflavonoids, and fixed oils and is used in folk medicine, mainly for wound healing and relief of inflammation and infectious diseases. It was observed that* O. fieldingiana* extracts are important source of antioxidants. It was possible to isolate a biflavonoid among the more polar constituents of the leaf extracts, while from the seeds a flavonoid glycoside, kaempferol 3-*O*-rutinoside, was identified, which when tested in association with known antimicrobial agent demonstrated superior antifungal action in relation to all other tested materials.

The* in silico* findings revealed that the compound amentoflavone coupled with the CYP450 protein due to the low energy stabilization, indicating a possible mechanism of action by inhibition of the ergosterol biosynthesis of* Candida* fungi.

## Figures and Tables

**Table 1:** Phenol and flavonoids content and antioxidant activity of *O. fieldingiana* extracts.

Extracts and constituents	**Total Phenols **	**Flavonoids **	**DPPH IC**_**50**_ ** (** **µ** **g/mL)**	**ABTS**
	**(mg GAE/g)** ^**∗**^	**(mg QE/g)** ^**∗∗**^		**(** **µ** **g/mL)**
**LEE**	35.33 ± 22.15^b^	8.978 ± 0.267^a^	4.953 ± 0.884^a^	5.117±2.605^a^
**LEE (HF)**	16.03 ± 14.29	3.798 ± 0.284	64.345 ± 0.227	63.210±1.577^d^
**LEE (CF)**	12.70 ± 8.38	1.261 ± 0.929	72.436 ± 0.359	59.613±1.916^d^
**LEE (EAF)**	23.30 ± 16.93^b^	5.216 ± 0.523	57.147 ± 2.085	9.654±0.161^b^
**LEE (MF)**	26.89 ± 9.46^a^	8.657 ± 0.195^a^	5.394 ± 0.03^a^	5.235±0.195^a^
**BEE**	61.81 ± 7.15^a^	7.225 ± 0.779^a^	5.898 ± 0.291^a^	4.195±0.0283^a^
**BEE (HF)**	9.60 ± 3.39	1.517 ± 0.241	310.486 ± 34.743	194.213±3.418^d^
**BEE (CF)**	8.013 ± 7.403	1.259 ± 0.094	108.386 ± 4.653	31.753±2.386^d^
**BEE (EAF)**	32.07 ± 1.29^b^	4.283 ± 0.123	7.577 ± 0.216^a^	6.166±0.164^a^
**BEE (MF)**	13.69 ± 6.99	0.979 ± 0.198	63.433 ± 3.456	62.455±0.018^d^
**SEF**	7.344±1.1482	0.229±0.0721	31.324±6.289	23.898±1.036^c^
**SEE**	6.007±3.007	0.688±0.095	71.092±1.116	27.676±0.467^c^
**SAE**	46.274±19.6^a^	5.849±0.296	16.096±0.636	64.228±0.298^d^
**Amentoflavone**	-	-	35.612±2.440	83.306±2.6354^d^
**Kaempferol 3-*O*-rutinoside**	-	-	30.962±1.5317	75.752±6.082^d^
**Quercetin**	-	-	4.779 ± 0.507^a^	1.738±0.089^a^

^*∗*^Total phenols are quantified in milligrams per gallic acid equivalent. ^*∗*^Flavonoids are quantified in milligrams per quercetin equivalent. ^*∗*^Confidence interval: 95%; LEE: leaf ethanol extract (LEE), BEE: branch ethanol extract, SEF: seed ethanol fraction, SEE: seed ethanol extract, and SAE: seed aqueous extract. HE: hexane fraction, CF: chloroform fraction, EAF: ethyl acetate fraction, and MF: methanol fraction.

**Table 2:** Evaluation of acetylcholinesterase inhibition action of extracts and constituents of *Ouratea fieldingiana.*

Extract	IC_50_ in ELISA (*µ*g/mL)
Leaf ethanol extract	0.816 ± 0.004^a^
Branch ethanol extract	11.89 ± 0.048^b^
Leaf ethanol extract (MF)	36.81 ± 0.024^d^
Branch ethanol extract (EAF)	57.58 ± 0.088^e^
Seed hexane extract	20.51 ± 0.387^c^
Seed ethanol extract	12.15 ± 0.003^b^
Seed aqueous extract	9.19 ± 0.030^b^
Kaempferol-3-*O*-rutinoside	17.70 ± 0.030^c^
Amentoflavone	11.92 ± 0.046^b^
Physostigmine (standard)	1.15 ± 0.046^a^
Eserine (standard; Penido et al., 2016)	19.53 ± 0.08^c^

Data presented are mean ± standard deviation, according to ANOVA followed by the Tukey test. Values with different small letters differ statistically (p<0.05); EAF: ethyl acetate fraction; MF: methanol fraction.

**Table 3:** Evaluation of the antifungal effect of *Ouratea fieldingina* extracts against *Candida* spp.

MIC 50/100 ^b^(*µ*g/mL)				
	Strains^a^			
Extracts	ATCC *C. parapsilosis *22019	ATCC *C. krusei *6258	*C.* *tropicalis* (R)	*C.* *parapsilosis* (R)
SHE	>1000 / >1000	>1000/>1000	>1000/>1000	>1000/ >1000
LEE	500 / > 1000	7.8 / 31.25	500 / > 1000	500 / > 1000
LEE (MF)	500 / > 1000	3.9 / 31.25	1000 / > 1000	125 / > 1000
BEE (EAF)	125 / 500	62.5 / 250	500 / > 1000	500 / > 1000
BEE	15.62 / > 1000	1.95 / 15.6	250 / > 1000	62.5 / > 1000
SEE	250 / 1000	62.5 / 250	1000 / > 1000	1000 / >1000

^a^Yeast strains isolated from collection. ^b^MIC was defined as the lowest concentration which produced 50% and 100% reduction of fungal cell growth after 24 h incubation. ^c^The procedure was performed according to protocol M27-A3 of CLSI 2008. The range of compounds tested varied from 1000 to 1.95 *μ*g/mL. SHE: seed hexane extract; LEE: leaf ethanol extract; LEE (MF): leaf ethanol extract (methanol fraction): BEE: branch ethanol extract, BEE (EAF): branch ethanol extract (ethyl acetate fraction), and SEE: seed ethanol extract.

**Table 4:** Evaluation of antifungal effect of amentoflavone and kaempferol against *Candida *spp. Isolates.

		**M** **I** **C**_50_^**b**^ **(** **µ** **g/mL)**		
	Strains^a^			
Compounds	ATCC *C. albicans *14053	ATCC *C. parapsilosis *22019	ATCC *C. krusei *6258	*Candida parapsilosis * (R)
Amentoflavone	125	15.62	15.62	250
Kaempferol 3-*O*-rutinoside	> 500	250	125	> 500
Amentoflavone + kaempferol 3-*O*-rutinoside	1.25	1.25	0.625	2.5

^a^Yeast cells isolated from collection. ^b^MIC was defined as the lowest concentration which produced 50% reduction of fungal cell growth after 24 h incubation. The procedure was performed according to protocol M27-A3 of CLSI 2008. The concentrations of compounds tested ranged from 500 to 0.97 *μ*g/mL.

## Data Availability

The data used to support the findings of this study are available from the corresponding author upon request.

## Additional Points

*Highlights.* (i) This study corroborates the traditional use for wound healing of* O. fieldingiana*. (ii) Amentoflavone was the main constituent of the plant leaf extract. (iii) The plant extracts displayed antioxidant and anticholinesterase activities. (iv) The antimicrobial activity was demonstrated in* in vitro* and* in silico* tests.

## Supplementary Materials

Supplementary Materials Graphical abstract figure shows the chemical compounds and biological actions of* Ouratea fieldingiana* (DC.) Baill.
