# Supplementary material for: Chemical Composition and Antifungal In Vitro and In Silico, Antioxidant, and Anticholinesterase Activities of Extracts and Constituents of Ouratea fieldingiana (DC.) Baill
Source: Evid Based Complement Alternat Med. 2018 Nov 7;2018:1748487. doi: 10.1155/2018/1748487 (PMC6247570; doi:10.1155/2018/1748487)
Supplement: Supplementary Materials — Graphical abstract figure shows the chemical compounds and biological actions of Ouratea fieldingiana (DC.) Baill. [file 1748487.f1.pdf]

Chemical compounds and biological actions of *Ouratea fieldingiana* (DC.) Baill

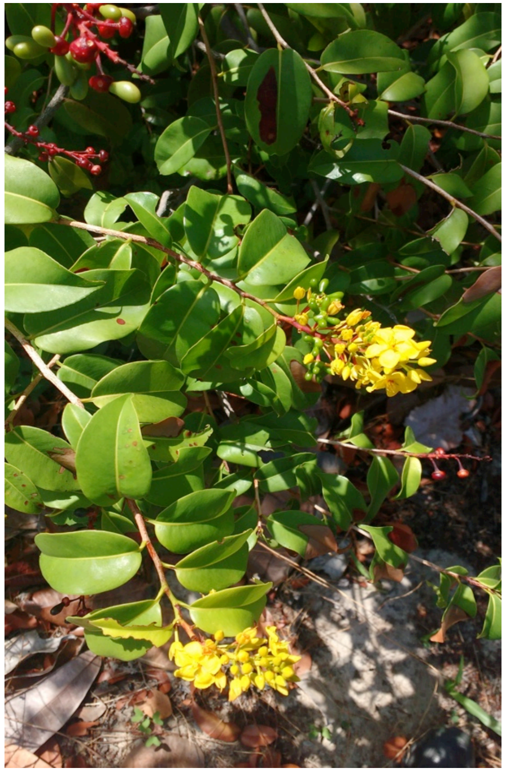

Ouratea fieldingiana

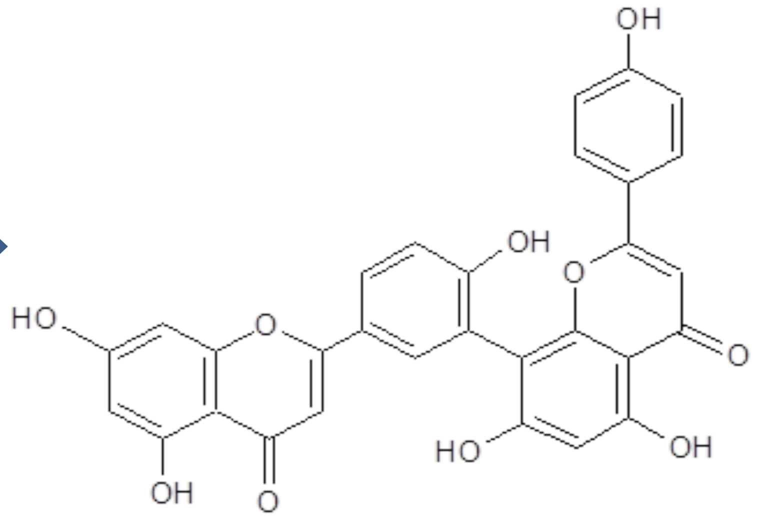

Amentoflavone

Activities:

- Wound healing
- Antifungal
- Inhibition of AChE
- Antioxidant

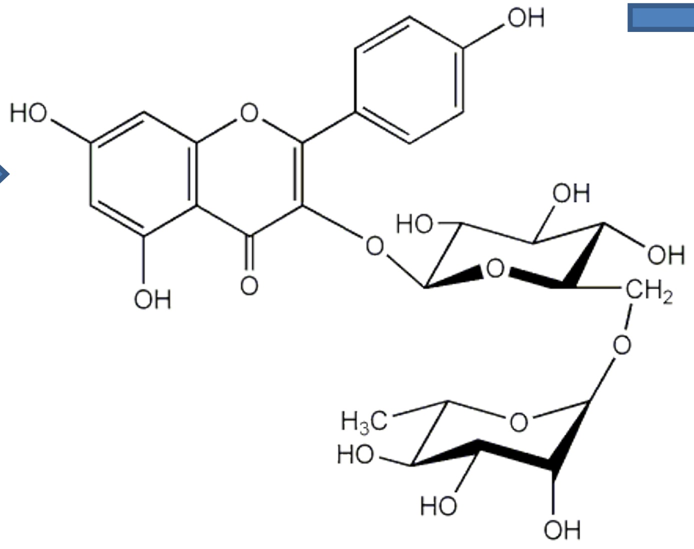

Kaempferol 3-*O*-rutinoside
